# Retraction Note: Evaluating polymicrobial immune responses in patients suffering from tick-borne diseases

**DOI:** 10.1038/s41598-022-07207-2

**Published:** 2022-02-21

**Authors:** Kunal Garg, Leena Meriläinen, Ole Franz, Heidi Pirttinen, Marco Quevedo-Diaz, Stephen Croucher, Leona Gilbert

**Affiliations:** 1 Department of Biological and Environmental Sciences, NanoScience Center, University of Jyväskylä, Jyväskylä, Finland; 2 Te?Ted Ltd, Mattilaniemi 6-8, Jyväskylä, Finland; 3 Institute of Virology, Biomedical Research Center, Slovak Academy of Sciences, Bratislava, Slovak Republic; 4 School of Communication, Journalism, and Marketing, Massey University, Wellington, New Zealand

Retraction of: *Scientific Reports* 10.1038/s41598-018-34393-9, published online 29 October 2018

The Editors have retracted this Article.

The main method utilised in this study is an ELISA assay. An investigation by the University of Jyväskylä, Finland, has concluded that the patient selection and description in this Article, and in an unpublished report validating the methods used, do not justify the results presented. The Editors therefore no longer have confidence in the results and conclusions presented in this Article.

Kunal Garg, Leena Meriläinen, Heidi Pirttinen, Marco Quevedo-Diaz, Stephen Croucher and Leona Gilbert disagree with this retraction. Ole Franz did not respond to correspondence from the Editors about this retraction.

